# Detection of influenza virus in rectal swabs of patients admitted in hospital for febrile illnesses in Thailand

**DOI:** 10.1177/2050312121989631

**Published:** 2021-01-22

**Authors:** Artit Sangkakam, Pasin Hemachudha, Abhinbhen W Saraya, Benjamard Thaweethee-Sukjai, Thaniwan Cheun-Arom, Alice Latinne, Kevin J Olival, Supaporn Wacharapluesadee

**Affiliations:** 1 Department of Internal Medicine, Loei Hospital, Loei, Thailand; 2 Thai Red Cross Emerging Infectious Diseases–Health Science Centre, World Health Organization Collaborating Centre for Research and Training on Viral Zoonoses, Chulalongkorn Hospital, Faculty of Medicine, Chulalongkorn University, Bangkok, Thailand; 3 Department of Biology, Faculty of Science, Ramkhamhaeng University, Bangkok, Thailand; 4 EcoHealth Alliance, New York, NY, USA; 5 Wildlife Conservation Society, Viet Nam Country Program, Ha Noi, Viet Nam; 6 Wildlife Conservation Society, Health Program, Bronx, NY, USA

**Keywords:** Diagnosis, fever, influenza, nasopharyngeal, rectal

## Abstract

**Introduction:**

Influenza virus favours the respiratory tract as its primary site of host entry and replication, and it is transmitted mainly via respiratory secretions. Nasopharyngeal swab is the gold standard specimen type for influenza detection, but several studies have also suggested that the virus replicates in the human gastrointestinal tract.

**Methods:**

A retrospective study was conducted on all patients positive for influenza virus and initially recruited as part of the PREDICT project from 2017 to 2018. The objectives of the study were to investigate whether rectal swab could aid in improving influenza detection, and if there was any correlation between gastrointestinal disturbances and severity of infection, using length of hospital stay as an indicator of severity.

**Results:**

Of the 51 influenza-positive patients, 12 had detectable influenza virus in their rectal swab. Among these 12 rectal swab positive patients, influenza virus was not detected in the nasopharyngeal swab of three of them. Gastrointestinal symptoms were observed for 28.2% patients with a negative rectal swab negative and 25.0% patients with a positive rectal swab. Average length of hospital stay was 4.2 days for rectal swab positive group and 3.7 days for rectal swab negative group. This difference was not statistically significant (p = 0.288).

**Conclusions:**

There is no correlation between influenza virus detection in rectal swab and gastrointestinal disturbances or disease severity, and there is currently insufficient evidence to support replicative ability in the gastrointestinal tract.

## Introduction

Influenza viruses are enveloped, negative single stranded RNA viruses. There are four types of influenza viruses, named A, B, C and D and classified under the Orthomyxoviridae family.^1^ Influenza C virus affects human and swine, usually causing mild to moderate respiratory infection, however, it is uncommon.^2^ Influenza D virus primarily affects cattle and is not known to cause infection in humans but is believed to have the potential to do so.^1,3^ Influenza A and B viruses are mainly responsible for seasonal flu. Influenza A virus (IAV) is known to cause pandemics, as animals can also be its natural reservoir, whereas influenza B virus (IBV) primarily infects humans.^4^

Influenza infection in humans can be mild to severe depending on the seasonal variation and the patient’s immune status. Typical upper respiratory tract symptoms with rhinorrhoea and pharyngitis accompanied by malaise, fever, myalgia and headaches are common. Gastrointestinal (GI) symptoms such as diarrhoea and/or vomiting does occur, and are usually more common in young children.^5,6^

Availability of molecular detection methods for influenza virus in recent years has improved diagnostic efficiency. Performance of polymerase chain reaction (PCR)-based tests indicated that nasopharyngeal flocked swabs should be regarded as the most sensitive samples for diagnosing influenza virus, with 94% sensitivity.^7,8^ Some studies have also reported detection of influenza viruses’ RNA in rectal swabs of patients with GI symptoms or severe systemic manifestations.^9,10,11^

The aim of this study was to explore whether faecal specimens or rectal swabs provided additional value in detecting influenza virus infection or predicting the severity of influenza infection by using duration of hospital stay as an indicator of severity. The study also aimed to investigate the relationship, if any, between influenza virus detected in rectal swabs and the patient’s corresponding GI symptoms. This study retrospectively analysed data collected as part of the PREDICT USAID project, a disease surveillance effort to strengthen early detection of viruses from animals and humans. All samples in this study were collected at Loei provincial hospital, Loei province, Thailand and sent to the Thai Red Cross Emerging Infectious Diseases Health Science Centre, Chulalongkorn University Hospital, Bangkok, Thailand, for viral screening, and influenza virus detection and characterization.

## Methods

### Clinical specimens

Local institutional review board approval number was obtained (IRB No. 380/59). Written informed consent was obtained from all individual participants enrolled in the study. For minor participants, written informed consent was also obtained from their parent or legal guardian. Questionnaire used in this study was approved and validated by the local IRB committee. All procedures performed in this study involving human participants were in accordance with the ethical standards of the institutional and/or national research committee, and with the 1964 Helsinki declaration and its later amendments or comparable ethical standards. Clinical samples from patients with febrile illness with undiagnosed cause of disease, such as severe acute respiratory disease, encephalitis of unknown origin and haemorrhagic fevers were obtained from Loei provincial Hospital in the north east of Thailand. The inclusion criteria were patient with febrile illness (more than 2 years old) and temperature > 37.5^o^C for less than 10 days. The exclusion criteria were patient who were unable to provide informed consent, specimen and/or disease history.

The sample size calculation was based on the prevalence of febrile illness at which hypothesized % frequency of outcome factor in the population was 0.716% at 95% confidence level. The equation for calculating sample size is shown below

$$N=\frac{z_{\propto /2}^{2}P(1-P)}{d^{2}}$$

Where N is sample size, Z^2^ is *Z* score (1.96 for 95% confidence level), P is hypothesized % frequency of outcome factor in the population (0.716% in this study), d is confidence limits: 5%, and N = 102.14 ~ 102 samples per year

Nasopharyngeal (NP) swab, rectal swab, urine and whole blood were collected from each hospitalized patient enrolled in the PREDICT project within 2 days of admission. The NP swabs and rectal swabs were collected from patients using flocked swabs (Copan Flock Technologies SRL, Italy) and rayon-tipped swabs, respectively (Puritan, USA). All samples were collected in viral transport medium. Total nucleic acids were extracted from the NP swab, rectal swab, and whole-blood using NUCLISENS^®^ EASYMAG^®^ automated method according to the manufacturer’s protocol.

PB1 gene was used to identify the influenza virus in NP swab, rectal swab and whole blood using reverse transcription polymerase chain reaction (RT-PCR). cDNA was synthesized with 8 µl of total nucleic acid using SuperScript™ III Reverse Transcriptase (Thermo Fisher Scientific, Waltham, Massachusetts, USA) according to the manufacturer’s protocol. Nested-PCR targeting the PB1 gene of influenza virus was performed with the primer sets FLUAPB1-F (ATGATGATGGGNATGTTYAAYATG) and FLUAPB1-R: (GCNGGNCCNAKDTCRYTRTTDATCAT) for the first round of PCR; and FLUAPB1-NF (GATGGGNATGTTYAAYATGYTDAGYAC) and FLUAPB1-R for the second round of PCR. This protocol was developed at the Centre for Infection and Immunity, Columbia University Mailman School of Public Health (Liang, E. Unpublished). Each PCR reaction included 2 µL of the cDNA for the first round of PCR or 2 µL of the first round PCR product for the second round of PCR in 50-µL reaction. Bands from the second round PCR product were visualized using the QIAxcel Advanced system and QIAxcel ScreenGel Software version 1.5.0 (QIAGEN, Hilden, Germany). RNAse/DNAse free water was used instead of template as negative control to exclude sample contamination during laboratory procedures and synthetic oligonucleotide was used as positive control. The final PCR products, with approximately 402 bp amplicon size, were agarose gel purified using NucleoSpin Gel and PCR Clean-up kit (MACHEREY-NAGEL, Germany) following the manufacturer’s instructions. The purified amplicons were then submitted for DNA sequencing by Sanger sequencing process using FLUAPB1-F and FLUAPB1-NF primers at First BASE Laboratories Sdn Bhd, Malaysia.

### Full length amplification for the influenza virus subtyping

Subtypes of all positive influenza virus samples were confirmed by identifying HA and NA gene sequences using specific primers and conventional RT-PCR protocol for human influenza surveillance (Table 1). The details of gene-specific primer sets for HA and NA genes of each type/subtype are shown in Table 1. Five µL of total nucleic acid was amplified in 50-μL reaction using one-step RT-PCR kit (Qiagen) following the manufacturer’s protocol. The PCR amplicon was separated by TBE agarose gel electrophoresis technique and purified using NucleoSpin Gel and PCR Clean-up kit (MACHEREY-NAGEL, Germany). The purified amplicon was sequenced (Sanger sequencing process) by First BASE Laboratories Sdn Bhd, Malaysia. To obtain full length sequence, a subset of gene fragment sequences for each type/subtype were aligned using BioEdit software version 7.0.5.3.

**Table 1.** Oligonucleotide primers designed for typing and subtyping of influenza viruses (IAV and IBV).

Type/subtype	Gene fragment	Primer	Sequence (5’ to 3’)	PCR product size (bp)
Influenza A H1N1 (2009)	HA-5’ (H1)	H1 F1	AGCAAAAGCAGGGGAAAATAAAAGC	1264
		H1R1264	CCTACTGCTGTGAACTGTGTATTC	
	HA-3’ (H1)	H1F848	GCAATGGAAAGAAATGCTGGATCTG	945
		HARUc	ATATCGTCTCGTATTAGTAGAAACAAGGGTGTTTT	
	NA-5’ (N1)	N1 F1	AGCAAAAGCAGGAGTTTAAAATG	1099
		N1R1099	CCTATCCAAACACCATTGCCGTAT	
	NA-3’ (N1)	N1F401	GGAATGCAGAACCTTCTTCTTGAC	1073
		NARUc	ATATGGTCTCGTATTAGTAGAAACAAGGAGTTTTTT	
Influenza A (H3N2)	HA-5’ (H3)	H3A1 F6	AAGCAGGGGATAATTCTATTAACC	1079
		H3A1R1	GTCTATCATTCCCTCCCAACCATT	
	HA-3’ (H3)	H3A1 F3	TGCATCACTCCAAATGGAAGCATT	863
		HARUc	ATATCGTCTCGTATTAGTAGAAACAAGGGTGTTTT	
	NA-5’ (N2)	NAFUc	TATTGGTCTCAGGGAGCAAAAGCAGGAGT	1095
		H3N2R1095	TCATTTCCATCATCRAAGGCCCA	
	NA-3’ (N2)	N2F387	CATGCGATCCTGACAAGTGTTATC	1082
		NARUc	ATATGGTCTCGTATTAGTAGAAACAAGGAGTTTTTT	
Influenza B	HA-5’	BHAF1u	TATTCGTCTCAGGGAGCAGAAGCAGAGCATTTTCTAATATC	1361
		BHAR1341	TTCGTTGTGGAGTTCATCCAT	
	HA-3’	BHAF458	AGAAAAGGCACCAGGAGGACCCTA	1391
		BHA2R1	GTAATGGTAACAAGCAAACAAGCA	

PCR: polymerase chain reaction.

### Clinical analysis

We identified all patients with samples positive for influenza virus and attempts were made to retrieve all in-patient medical records. We categorized these patients into two groups, one with NP swab positive and one with rectal swab positive regardless of NP results. Information for all patients were retrieved from the PREDICT surveillance project questionnaire, which was administered to all patients enrolled in the study and included demographic details, temperature at presentation, symptoms, and initial treatments.

Furthermore, we were able to obtain 39 out of 51 in-patient medical records and their laboratory work up were analysed, and length of hospital stay, maximum body temperature during hospital stay, white cell counts, and GI symptoms (defined as either vomiting and/or diarrhoea) were collected.

We were unable to specify duration from onset of symptoms to sample collection as in-patient details were not adequate and did not specify initial symptoms, which could range from prodromal flu-like to fever.

### Statistical analysis

A Mann-Whitney U test was used to compare differences in length of hospital stay, maximum temperature during hospital stay and white cell counts between patients with positive rectal swab and those with a negative rectal swab. All statistical analyses were performed using SPSS Statistics 17.0.

## Results

**Figure 1.**
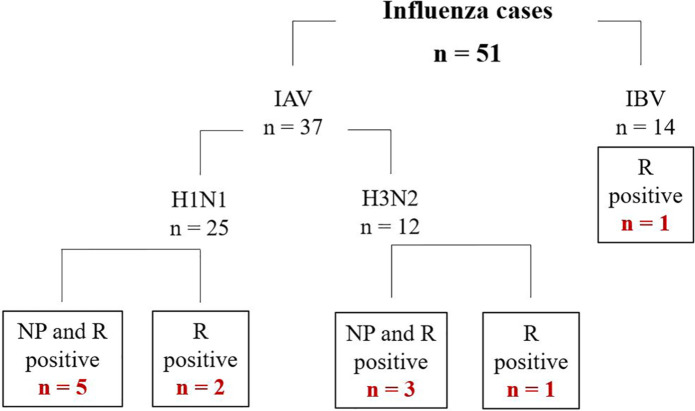
Summary of nasopharyngeal (NP) and rectal (R) swab findings. Subtyping in cases with both samples positive were consistent.

A total of 200 human participants were enrolled in this study from May 2017 through to December 2018 with 51 influenza positive cases detected via PCR. NP swab was positive for 48 of 51 cases. Rectal swab was positive for 12 of 51 cases. Within the positive rectal swab group, three cases were isolated rectal swab positive as their NP swab was negative. Influenza virus typing results are summarized in Figure 1. IAV represented 37 of 51 cases, 25 samples were H1N1 and 12 samples with H3N2. Within IAV group, a total of 11 rectal swabs were positive (8 NP and rectal positives and 3 isolated rectal positives). IBV represented the remaining 14 of 51 cases with only 1 patient with isolated rectal swab positive. None of the whole blood samples from these 51 patients were positive for Influenza. All 12 patient’s medical records with positive rectal swab were obtained and their demographics, clinical features, and virological information are summarized in Table 2. We were also able to obtain medical records from 27 of 39 patients with positive NP swab but negative rectal swab.

**Table 2.** Demographics, virological data and clinical features of 12 patients tested positive for influenza virus in their rectal swabs.

Patient	Sex/age (year)	Stool	Co-morbidities	Presenting symptoms	Virus typing	Hospital stay (days)	Temp (°C)	White Cell Count (per µL)
**1**	M/4	Firm	None documented	Fever, cough, vomit, reduced appetite	IAV (H3 N2)	2	39.4	10,730
**2**	M/7	Firm	None documented	Fever, cough	IBV	3	39.7	6160
**3**	M/3	Firm	None documented	Fever, injected conjunctiva, cough, myalgia, vomit, reduced appetite	IAV (H1N1)	7	40	9910
**4**	F/2	Watery	None documented	Fever, cough, rhinorrhoea, diarrhoea, reduced appetite	IAV (H1N1)	3	40.2	3680
**5**	M/17	Firm	Smoker	Fever, headache, rigour, confusion	IAV (H1 N1)	6	38.7	5190
**6**	F/19	Firm	None documented	Fever, cough, rhinorrhoea, headache, myalgia, fatigue	IAV (H1N1)	5	38.7	3840
**7**	F/32	Firm	None documented	Fever, cough, headache, myalgia, fatigue	IAV (H1N1)	3	40.6	8200
**8**	M/32	Firm	Cellulitis	Fever	IAV (H3N2)	4	39.6	19,150
**9**	M/31	Firm	None documented	Fever, abdominal pain, headache, myalgia, fatigue	IAV (H3N2)	3	39.3	10,100
**10**	F/23	Firm	None documented	Fever, cough, myalgia	IAV (H1N1)	6	39.5	5530
**11**	F/61	Firm	None documented	Fever, cough, headache, back pain	IAV (H1 N1)	4	40.2	4670
**12**	M/21	Firm	None documented	Fever, shortness of breath, headache, rigour, myalgia, fatigue	IAV (H3N2)	4	40.5	11,680

IAV: influenza A virus; IBV: influenza B.

None of the patients included in this study required inotropic support, intensive care, or mechanical ventilation, and no mortality was observed. There was no statistically significant difference in the length of hospital stay for patients with positive rectal swab (n = 12, mean ± SD = 4.2 ± 1.5) compared to the negative rectal swab group (n = 27, mean ± SD = 3.7 ± 1.1), Mann–Whitney Test (Z = -1.063, p = 0.288). Mean peak white cell counts of patients with positive rectal swab (n = 12, mean ± SD = 8237 cells/µL ± 4443) was compared to those of patients with negative rectal swab (n = 27, mean ± SD = 8152 cells/µL ± 4728), Mann–Whitney Test (Z = -0.228, p = 0.818). Mean peak temperature of patients with positive rectal swab (n = 12, mean ± SD = 39.7°C ± 0.63) and patients with negative rectal swab (n = 27, mean ± SD = 39.5°C ± 0.74) were also compared, Mann–Whitney Test (Z = -0.669, p = 0.503). There was no statistically significant difference in mean peak white cell counts and peak temperature during hospital stay for both groups.

GI symptoms were observed in 11/39 patients with positive NP swab and negative rectal swab (28.2%), whereas GI symptoms were seen in 3/12 patients with positive rectal swab (25.0%). Within the positive rectal swab group, one patient (patient 4) was documented to have watery stool during admission, while two patients (patients 1 and 3) were vomiting before admission. There is no documentation of GI symptoms before or during admission for the remaining rectal swab positive patients (Table 2).

## Discussion

A study by Sato et al.^12^ suggested that influenza virus cannot replicate in the GI tract as the acidity would render it non-infectious. However, the use of acid lowering therapies and the possibility of virus mixing with food debris may reduce acid exposure and help the virus maintain its replicative ability in the GI tract.^12^ Hirose et al.^13^ (2017) performed colonoscopy in patients with influenza virus infection, which demonstrated enteritis and biopsy showing presence of influenza RNA and antigens.^13,14^ However, detectable viral RNA in rectal swab does not confirm viral replication in the GI tract as there are no surface receptors on human intestinal epithelial cells compatible with influenza virus replication. Influenza RNA detection in the GI tract is therefore more likely to result from ingestion of mucus containing viral RNA.^15^ On the other hand, Shu et al.^16^ suggested the possibility that severe infection can lead to detectable viral RNA in the GI tract due to dissemination of lymphocytes into the tract, and that this systemic inflammation could be responsible for enteritis.^17^

Our study found no correlation between GI disturbance and presence of influenza virus RNA in the GI tract, as similar proportion of patients experienced GI symptoms in the group with positive NP swab and negative rectal swab and in the group of patients with positive rectal swab. There was no difference in the duration of hospital stay, white cell count and temperature between the two groups. A recent study found saliva, a more convenient and less invasive specimen, to be almost as sensitive for influenza virus screening as NP swab.^18^ Therefore, rectal swab testing for the sole purpose of detection of influenza may add little to the diagnoses and treatment of the patient.

Limitation of this study includes its retrospective nature, as we were not able to obtain the time interval between the onset of fever and the rectal swab sample collection. We cannot exclude that the isolated positive rectal swabs observed in this study could be due to the persistence of influenza RNA in the faecal sample despite viral eradication from respiratory samples.^9^ It is also possible that negative NP swab of these patients was due to inadequacy in NP collection. As we were not able to obtain all medical records, the length of hospital stay, temperature and white cell count were calculated from 39/51 patients.

## Conclusion

We have shown that rectal swabs cannot be used to predict the severity of influenza infection. Presence of detectable virus were not associated with GI disturbance, and there is currently no evidence to suggest viral replication in the intestine. In some cases, it is possible to have an isolated positive rectal swab but systematic rectal swab testing would only provide minimal diagnostic benefit and is unlikely to be cost effective.

## Supplemental Material

sj-docx-1-smo-10.1177_2050312121989631 – Supplemental material for Detection of influenza virus in rectal swabs of patients admitted in hospital for febrile illnesses in Thailand Click here for additional data file. Supplemental material, sj-docx-1-smo-10.1177_2050312121989631 for Detection of influenza virus in rectal swabs of patients admitted in hospital for febrile illnesses in Thailand by Artit Sangkakam, Pasin Hemachudha, Abhinbhen W Saraya, Benjamard Thaweethee-Sukjai, Thaniwan Cheun-Arom, Alice Latinne, Kevin J Olival and Supaporn Wacharapluesadee in SAGE Open Medicine

sj-png-2-smo-10.1177_2050312121989631 – Supplemental material for Detection of influenza virus in rectal swabs of patients admitted in hospital for febrile illnesses in Thailand Click here for additional data file. Supplemental material, sj-png-2-smo-10.1177_2050312121989631 for Detection of influenza virus in rectal swabs of patients admitted in hospital for febrile illnesses in Thailand by Artit Sangkakam, Pasin Hemachudha, Abhinbhen W Saraya, Benjamard Thaweethee-Sukjai, Thaniwan Cheun-Arom, Alice Latinne, Kevin J Olival and Supaporn Wacharapluesadee in SAGE Open Medicine
