# Supplementary material for: Detection of influenza virus in rectal swabs of patients admitted in hospital for febrile illnesses in Thailand
Source: SAGE Open Med. 2021 Jan 22;9:2050312121989631. doi: 10.1177/2050312121989631 (PMC7841862; doi:10.1177/2050312121989631)
Supplement: sj-docx-1-smo-10.1177_2050312121989631 – Supplemental material for Detection of influenza virus in rectal swabs of patients admitted in hospital for febrile illnesses in Thailand [file sj-docx-1-smo-10.1177_2050312121989631.docx]

**Author declaration for ‘Detection of influenza virus in rectal swabs of patients admitted in hospital for febrile illnesses in Thailand’**

1. **Ethics approval**

Ethical approval for this study was obtained from Faculty of Medicine, Chulalongkorn University (IRB No. 380/59). All procedures performed in this study involving human participants were in accordance with the ethical standards of the institutional and /or national research committee, and with the 1964 Helsinki declaration and its later amendments or comparable ethical standards.

1. **Informed consent**

Written informed consent was obtained from all individual participants enrolled in the study. For minor participants, written informed consent was also obtained from their parent or legal guardian.

1. **Trial registration**

Not applicable.

Pasin Hemachudha, Chulalongkorn University, Phyathai Road, Bangkok 10330, Thailand. Email: [pasin.hemachudha@gmail.com](mailto:pasin.hemachudha@gmail.com)
