# Supplementary figures and images for: Detection of influenza virus in rectal swabs of patients admitted in hospital for febrile illnesses in Thailand
Source: SAGE Open Med. 2021 Jan 22;9:2050312121989631. doi: 10.1177/2050312121989631 (PMC7841862; doi:10.1177/2050312121989631)

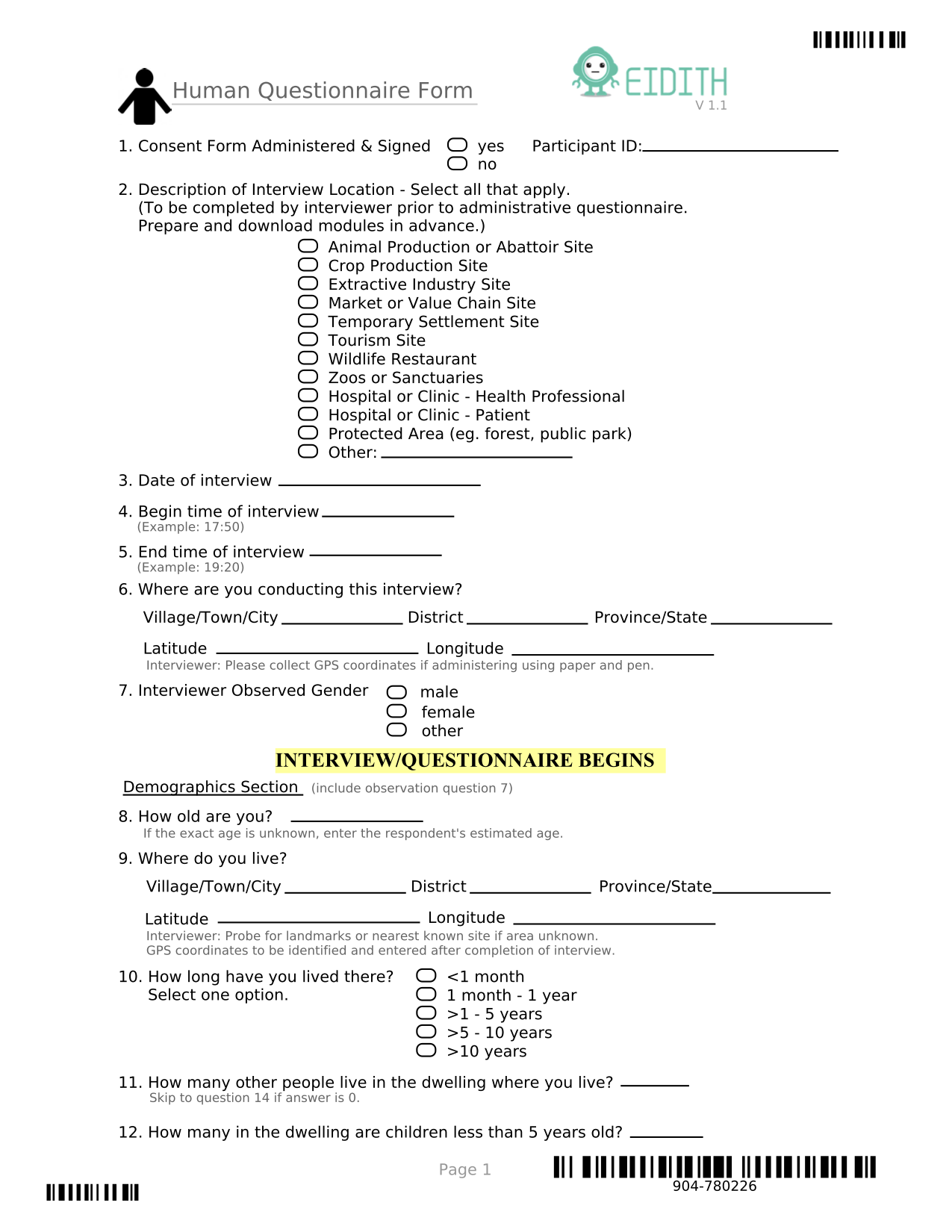

Supplement: sj-png-2-smo-10.1177_2050312121989631 – Supplemental material for Detection of influenza virus in rectal swabs of patients admitted in hospital for febrile illnesses in Thailand [file sj-png-2-smo-10.1177_2050312121989631.png]
